# The Application of Inosine 5′-Monophosphate Dehydrogenase Activity Determination in Peripheral Blood Mononuclear Cells for Monitoring Mycophenolate Mofetil Therapy in Children with Nephrotic Syndrome

**DOI:** 10.3390/ph13080200

**Published:** 2020-08-18

**Authors:** Joanna Sobiak, Alicja Jóźwiak, Honorata Wziętek, Jacek Zachwieja, Danuta Ostalska-Nowicka

**Affiliations:** 1 Department of Physical Pharmacy and Pharmacokinetics, Poznan University of Medical Sciences, 60-781 Poznań, Poland; alicja_joz7@wp.pl (A.J.); honorata.wzietek1@gmail.com (H.W.); 2 Department of Pediatric Nephrology and Hypertension, Poznan University of Medical Sciences, 60-572 Poznań, Poland; jacekzachwieja@ump.edu.pl (J.Z.); dostalska@ump.edu.pl (D.O.-N.)

**Keywords:** mycophenolic acid, inosine-5′-monophosphate dehydrogenase activity, pediatric patients, HPLC method, therapeutic drug monitoring, nephrotic syndrome, pharmacokinetics, pharmacodynamics

## Abstract

In pediatric nephrotic syndrome, recommended mycophenolic acid (MPA) pharmacokinetics are higher than those for transplant recipients. In MPA therapeutic monitoring, inosine-5′-monophosphate dehydrogenase (IMPDH) activity may be useful. We modified the method established for renal transplant recipients and determined IMPDH activity in peripheral blood mononuclear cells (PBMCs) from healthy volunteers and children (4–16 years) with nephrotic syndrome treated with mycophenolate mofetil (MMF). From children, four blood samples were collected, and MPA concentrations were also determined. IMPDH activity was calculated using xanthosine monophosphate (XMP) normalized with adenosine monophosphate (AMP), both determined with the HPLC-UV method. The modified method was accurate, precise, and linear for AMP and XMP within 0.50–50.0 μmoL/L. Mean IMPDH activity in volunteers was 45.97 ± 6.24 µmoL·s^−1^·moL^−1^ AMP, whereas for children, the values were variable and amounted to 39.23 ± 27.40 µmoL·s^−1^·moL^−1^ AMP and 17.97 ± 15.24 µmoL·s^−1^·moL^−1^ AMP before the next MMF dose and 1 h afterward, respectively. The modified method may be applied to IMPDH activity determination in children with nephrotic syndrome treated with MMF. IMPDH activity should be determined after one thawing of PBMCs due to the change in AMP and XMP concentrations after subsequent thawing. For children, the lowest IMPDH activity was observed concomitantly with the highest MPA concentration.

## 1. Introduction

Immunosuppressive drugs aim to decrease immune system activity and inhibit inflammatory processes. The ideal immunosuppressant should act selectively on immune system reactions and consequently present low toxicity. So far, no immunosuppressant is devoid of adverse effects, as these drugs act on quickly proliferating cells. One of the most frequently administered drugs after solid organ transplantation is mycophenolate mofetil (MMF), the active form of which, mycophenolic acid (MPA), inhibits the activity of inosine 5′-monophosphate dehydrogenase (IMPDH) [1,2]. MMF is more effective, reduces the severity of graft rejection episodes, and influences the overall survival of patients and, therefore, has replaced azathioprine [3].

MPA inhibits IMPDH activity expressed in activated T and B lymphocytes. IMPDH is responsible for de novo purine synthesis and depends on nicotinamide adenine dinucleotide (NAD^+^). IMPDH catalyzes the oxidation of inosine-5′-monophosphate (IMP) to xanthosine-5′-monophosphate (XMP), which subsequently changes into guanosine-5′-monophosphate (GMP) and converts to guanosine-5′-triphosphate (GTP) and deoxyguanosine triphosphate (dGTP). GTP and dGTP are essential nucleotides for DNA and RNA synthesis [4,5]. As a consequence, by inhibiting IMPDH activity in a selective, reversible, and noncompetitive manner, MPA leads to a decrease in guanine nucleotide synthesis and reduces cell proliferation. This mechanism is responsible for MPA’s favorable anticancer, immunosuppressive, and antiviral activity [6].

Significant inter- and intrapatient variability of MPA pharmacokinetics provides the basis for therapeutic drug monitoring (TDM) in the case of MMF; however, its importance is still being discussed [1]. In the literature, studies of MPA pharmacokinetics concern mainly children after transplantation [7,8,9,10,11]. However, in our previous study [12], in children with nephrotic syndrome, we observed higher trough MPA concentration (C_trough_) and area under the time-concentration curve (AUC) if compared to recommended C_trough_ and AUC for transplant recipients [8]. This observation was later described also by other authors [13,14]. Determining IMPDH activity may serve as the pharmacodynamic marker of MPA as, according to the literature, determining IMPDH activity before transplantation should help MMF dose individualization [15]. Although this approach is promising [16,17,18], studies concerning IMPDH activity in relation to MPA concentrations are limited [15,19,20,21,22,23]. Moreover, according to our knowledge, there are no studies including IMPDH activity in children with nephrotic syndrome.

Due to the difference in MPA pharmacokinetics between children with nephrotic syndrome and renal transplant recipients, and the suggestions for introducing MPA TDM in children with nephrotic syndrome, we decided to verify whether the IMPDH activity values in children with nephrotic syndrome are within the range of that reported in the literature for other subjects. To achieve this aim, firstly, we modified and validated the HPLC-UV method of Glander et al. [24]. Secondly, we determined the IMPDH activity in peripheral blood mononuclear cells (PBMCs) from healthy volunteers. Thirdly, we determined the IMPDH activity in PBMCs and plasma MPA for children with nephrotic syndrome treated with MMF and compared the results with the literature data.

## 2. Results

### 2.1. The Modifications of the IMPDH Activity Determination Method

For determining IMPDH activity in PBMCs, we introduced some changes to Glander et al.’s study [24]. We checked a few method parameters: mobile phase (pH 5.2 and 5.6; buffer:methanol proportions of 92:8 and 90:10), NAD^+^ concentrations in the incubation buffer (0.5 and 1.0 mmoL/L), PBMC incubation time (120, 150, and 180 min), detection wavelength (254 and 260 nm), and the stability after freeze–thaw cycles.

**Figure 1:**
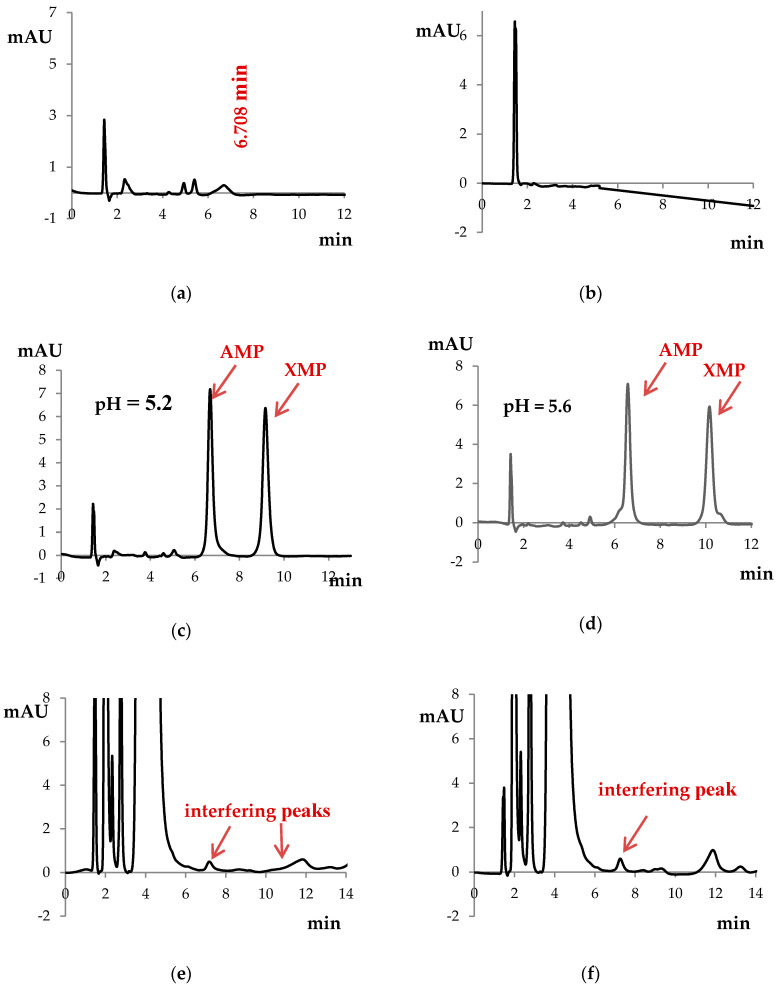
The HPLC chromatograms of (**a**) incubation buffer with bovine serum albumin (mobile phase buffer pH 5.2), (**b**) incubation buffer without bovine serum albumin (mobile phase buffer pH 5.2), (**c**) 10 µmoL/L of adenosine monophosphate (AMP) and 10 µmoL/L of xanthosine monophosphate (XMP) at mobile phase buffer pH of 5.2, (**d**) 10 µmoL/L of AMP and 10 µmoL/L of XMP at mobile phase buffer pH of 5.6, (**e**) incubation buffer containing 0.5 mmoL/L nicotinamide adenine dinucleotide (NAD^+^), and (**f**) incubation buffer containing 1.0 mmoL/L NAD^+^.

Initially, we used bovine serum albumin to prepare the incubation buffer for calibration curves, as adenosine monophosphate (AMP) and XMP occur in PBMCs, and blank samples of PBMCs cannot be used for calibration. However, we found an interfering peak around AMP retention time if bovine serum albumin was included. Therefore, we decided to use HPLC-grade water without bovine serum albumin for preparing the incubation buffer for calibration curves (Figure 1a,b).

To separate the analytes precisely, we checked different pHs of the mobile phase buffer (5.6 and 5.2). We observed the best separation and symmetry of peaks at pH 5.2 (Figure 1c,d). Therefore, pH 5.2 was used throughout the rest of the studies.

We checked the influence of different NAD^+^ concentrations (0.5 and 1.0 mmoL/L) on the AMP and XMP chromatographic peak appearances (Figure 1e,f).

Better symmetry and less blur were observed with 1.0 mmoL/L NAD^+^ in the incubation buffer. However, concomitantly, the analysis revealed the interfering peak around the retention time of AMP. For NAD^+^ at a concentration of 0.5 mmoL/L, two peaks were observed, whereas for 1.0 mmoL/L NAD^+^, one peak appeared. We verified whether any change in HPLC analysis parameters (detection wavelength 260 nm instead of 254 nm; mobile phase composition 92:8 instead of 90:10, *v/v*) could help reduce the peak surface area. We found the smallest peak surface area along with the best AMP and XMP peaks’ resolutions for detection wavelength 254 nm, and the mobile phase ratio of mixing 90:10 (*v/v*). Therefore, these conditions were used during HPLC analysis along with NAD^+^ 1 mmoL/L for sample preparation. The interfering peak surface area was reproducible; thus, the AMP surface area was reduced by the surface area of the interfering peak in the incubation buffer after each PBMC sample analysis. The mean surface area of the interfering peak for the above-described conditions was 8.208 ± 0.635 mAU∙s (*n* = 5) and did not exceed 10% of the AMP peak surface area in healthy volunteers.

### 2.2. The Validation of the AMP and XMP Determination Method

For AMP and XMP determination, the lower limit of quantification (LLOQ) was 0.50 μmoL/L. Both methods were linear within the range 0.50–50.0 μmoL/L, and the correlation coefficients were 0.9999 for both curves. The intercept value of both standard curves was significant, and the equations for calibration curves (*n* = 5) were as follows:

P_AMP_ = 11.9439 × C_AMP_−0.8319 (1)

P_XMP_ = 13.6073 × C_XMP_−1.6779 (2)

For AMP determination, within-run coefficient of variation (CV) and relative error (%RE) were within the 0.57–2.33% and −5.00 to 4.44% ranges, respectively. Between-run CV and %RE were within the 2.59–16.02% and −2.40 to 10.00% ranges, respectively. For XMP determination, within-run CV and %RE were within the 0.20–1.85% and −2.00 to 0.68% ranges, respectively. Between-run CV and %RE were within the 0.95–6.50% and −2.00 to 6.80% ranges, respectively.

Mean (±SD) retention times were 7.31 ± 0.06 min and 10.42 ± 0.19 min for AMP and XMP, respectively.

AMP and XMP concentrations were stable after three cycles of freeze–thaw. For AMP, %RE was within −2.74 to (−1.10%) and 10.00–18.00% for 50 and 1.0 μmoL/L, respectively. For XMP, %RE was within −0.04 to 1.06% and −3.00 to 9.00% for 50 and 1.0 μmoL/L, respectively.

For MPA concentrations of 0.5 and 20 μg/mL in both solvents (water and incubation buffer), we found no MPA peak within the retention times of AMP and XMP as well as up to 25 min.

### 2.3. IMPDH Activity in Healthy Volunteers

We determined the IMPDH activity after different incubation times (120, 150, and 180 min) in PBMC samples from three healthy volunteers. Mean AMP concentration did not differ between 120, 150, and 180 min of incubation; however, XMP concentration increased by 37% and 17% if the incubation was prolonged from 120 to 150 min, and from 150 to 180 min, respectively. Mean (±SD) IMPDH activity was 34.03 ± 9.02 µmoL∙s^−1^∙moL^−1^ AMP, 44.38 ± 13.00 µmoL∙s^−1^∙moL^−1^ AMP, and 46.42 ± 11.99 µmoL∙s^−1^∙moL^−1^ AMP for 120, 150, and 180 min, respectively. IMPDH activity increased within 23.64–34.89% after prolonging the incubation from 120 to 150 min, whereas, if the incubation was prolonged from 150 to 180 min, IMPDH activity increased within 0.52–14.82%. Worse symmetry of the XMP peak was observed if the incubation lasted 120 min; therefore, we incubated the samples for 150 min as in the Glander et al. study [24].

In PBMCs from healthy volunteers, we checked whether the concentration of NAD^+^ in the incubation buffer influences the determined IMPDH activity. Mean (±SD) IMPDH activity amounted to 56.26 ± 9.59 µmoL∙s^−1^∙moL^−1^ AMP (range, 42.85–69.13 µmoL∙s^−1^∙moL^−1^ AMP) and 45.97 ± 6.24 µmoL∙s^−1^∙moL^−1^ AMP (range, 37.53–54.14 µmoL∙s^−1^∙moL^−1^ AMP) for 0.5 and 1.0 mmoL/L NAD^+^, respectively. The difference was close to being significant (*p* = 0.052).

In PBMCs from three healthy volunteers, the stability after three cycles of freeze–thaw was assessed (Table 1). The results concerning AMP concentrations were not conclusive, as after the second thawing, the AMP concentration was stable in PBMCs from one volunteer and decreased in PBMCs from two volunteers. After the third thawing, the AMP concentration was further reduced in samples from two volunteers and increased in the sample from the volunteer with stable AMP concentrations after the second freeze–thaw cycle. XMP concentrations decreased by approximately 20% in all samples after the second freeze–thaw cycle; however, after the third cycle, the value increased in samples from two volunteers and decreased further in samples from one volunteer. Consequently, IMPDH activity in all samples decreased after the second cycle and increased in 2/3 of samples after the third cycle.

### 2.4. AMP and XMP Concentrations and IMPDH Activity in Children with Nephrotic Syndrome Treated with MMF

**Figure 2:**
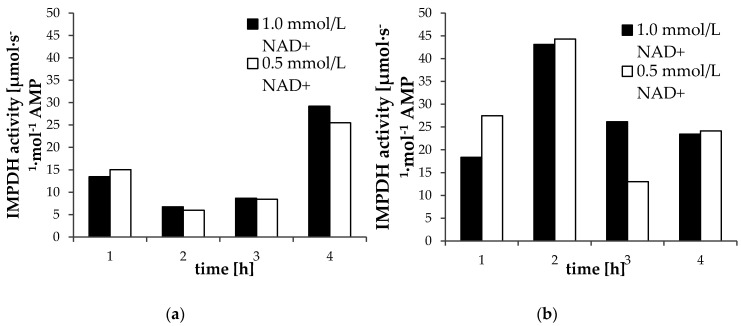
Inosine 5′-monophosphate dehydrogenase (IMPDH) activity in peripheral blood mononuclear cells (PBMCs) from child No. 2 (**a**) and No. 5 (**b**) after incubation with nicotinamide adenine dinucleotide (NAD^+^) at a concentration of 0.5 mmoL/L (white bar) and 1.0 mmoL/L (black bar).

We checked the influence of NAD^+^ concentrations (0.5 and 1.0 mmoL/L) in the incubation buffer on IMPDH activity in two children (Figure 2). In the first child, in three out of four samples, IMPDH activity was higher when NAD^+^ at a concentration of 1.0 mmoL/L was used. Such an observation was noted in only one out of four samples collected from the second child; however, for this child, we also observed an unexpected increase of the activity deteremined 1 h after drug administration (A_1_). With reference to the above-described results for samples preparation, for further analysis, NAD^+^ at a concentration of 1.0 mmoL/L was used.

**Figure 3:**
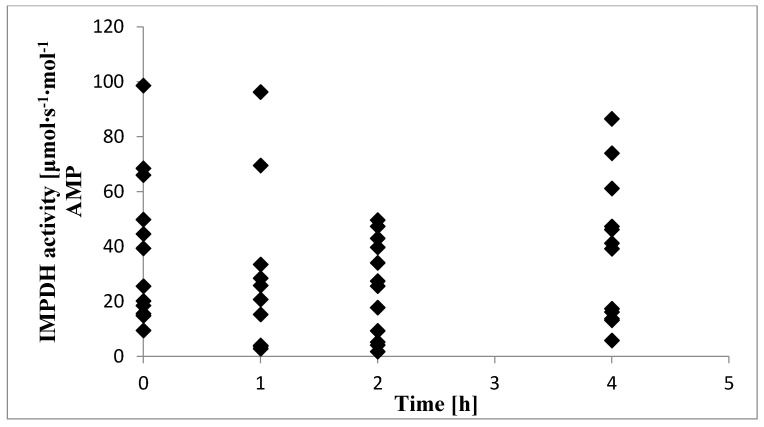
The individual values of inosine 5′-monophosphate dehydrogenase (IMPDH) activity in relation to time elapsed after mycophenolate mofetil (MMF) administration in 12 children with nephrotic syndrome.

For 12 children, we determined AMP and XMP concentrations and calculated IMPDH activity in PBMCs (Table 2) using established sample preparation procedures and HPLC conditions described previously. Median activity in the sample collected before the next drug dose administration (A_trough_) and 1 h (A_1_), 2 h (A_2_), and 4 h (A_4_) afterward were 32.4, 18.0, 26.5, and 40.2 µmoL∙s^−1^∙moL^−1^ AMP, respectively. The IMPDH activity within the first 4 h after MMF administration was variable, as CV% was within 68–115%. The highest variability was observed for A_1_. Mean A_trough_ was twofold higher than the minimal enzyme activity (A_min_). For all children included in the study, the individual values of IMPDH activity in relation to time elapsed after MMF administration are presented in Figure 3.

### 2.5. The Relation Between IMPDH Activity and MPA Concentrations in Children with Nephrotic Syndrome Treated with MMF

**Figure 4:**
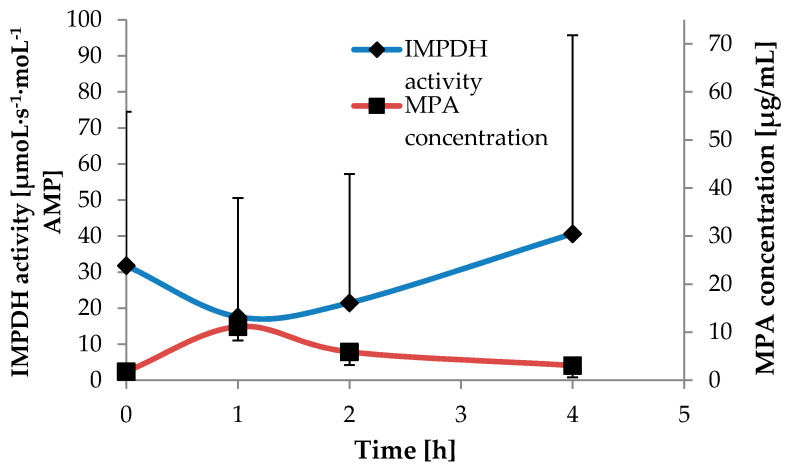
Inosine 5′-monophosphate dehydrogenase (IMPDH) activity and mycophenolic acid (MPA) concentration determined within 4 h after mycophenolate mofetil (MMF) administration.

The comparison between the mean calculated IMPDH activity and mean plasma MPA concentrations within 4 h after MMF administration is presented in Figure 4. Table 3 shows the pharmacodynamic and pharmacokinetic parameters. The highest IMPDH activity, along with the lowest MPA concentration, was observed in samples collected at time 0 (A_trough_ and C_trough_). The lowest IMPDH activity was A_1_, and at this time point, the MPA concentration was the highest and close to the MPA maximal concentration (C_max_). The IMPDH A_2_ was close to A_1_; however, MPA C_2_ was almost twofold lower than C_1_. The variability of IMPDH area under the effect–time curve from 0 to 4 h (AEC_0–4_) was twofold higher than the MPA area under the concentration–time curve from 0 to 4 h (AUC_0–4_), as CV amounted to 73% and 33%, respectively.

## 3. Discussion

MPA pharmacokinetics is highly variable due to numerous factors [1]. The recommended target values of MPA C_trough_ and AUC were established for renal transplant recipients [1,8]. However, MMF is currently more often administered also for other indications [16]. In our previous study [12], we found that MPA C_trough_ and AUC in children with nephrotic syndrome are higher than recommended target values for renal transplant recipients. As MPA inhibits the activity of IMPDH and there are some studies describing IMPDH activity in renal transplant recipients, we decided to verify whether it is possible to apply the method found in the literature [24] to calculate IMPDH activity in children with nephrotic syndrome treated with MMF. In our study, we checked a few aspects of sample preparation as well as a few parameters of the chromatographic analysis and verified the results in samples of PBMCs from healthy volunteers. Finally, we applied the method to determine IMPDH activity in children with nephrotic syndrome treated with MMF and compared the results with MPA plasma concentrations determined for these children within 4 h after MMF administration.

There are two kinds of methods for determining IMPDH activity: radiometric and nonradiometric. The first method is used to determine antimetabolites in intact human lymphocytes and allows determining intracellular MPA concentration. The second method is used for such matrices as erythrocytes, whole blood, and PBMCs [5], and this kind of method is described in Glander et al.’s study [24].

IMPDH activity may be normalized on cell count, protein concentration, or intracellular AMP concentration. In our study, IMPDH activity was normalized on AMP concentration as in the Glander et al. study [24]. The advantage of this approach is that it helps to minimalize the influence of impurities [23]. The disadvantage is that AMP may occur in commercial standards of IMP and NAD^+^ [25]. In our study, we found two interfering peaks for incubation buffer when NAD^+^ at a concentration 0.5 mmoL/L was used and one interfering peak for NAD^+^ at a concentration of 1.0 mmoL/L. We changed the detection wavelength, the mobile phase pH, and the ratio of mixing mobile phase solutions and concluded that we were unable to remove these peaks. In PBMC samples from healthy volunteers, the difference in IMPDH activity was not significant whether NAD^+^ at a concentration of 0.5 or 1.0 mmoL/L was used. Therefore, we chose the latter for sample preparation and diminished each AMP peak surface area by the surface area of the interfering peak from the incubation buffer.

Regarding Glander et al.’s study [24], we were unable to use bovine serum albumin in the incubation buffer, as we observed an interfering peak around AMP retention time in samples with bovine serum albumin. It is impossible to use blank samples as calibrators due to AMP and XMP natural occurrence in PBMCs [24]. We prepared the incubation buffer using HPLC-grade water.

The method was precise, accurate, and linear within the 0.5-50 μmoL/mL range for determining the concentrations of both analytes—AMP and XMP. In comparison to Glander et al.’s study [24], we determined lower AMP and XMP concentrations as the authors described the linearity of the method within the 0.75–50 μmoL/mL range. For AMP, lowering the limit of detection is not of the highest importance due to the occurrence of the interfering peak from the incubation buffer. This peak did not occur in samples for calibration curves. In our opinion, IMPDH activity should be determined in samples thawed only once due to the instability of AMP and XMP concentrations after the second and third freeze–thaw cycles. On the contrary, other authors proved the freeze–thaw stability of AMP and XMP concentrations after three cycles [25]. MPA did not interfere in AMP and XMP determination and should be determined in plasma using a different analytical method. It is in accordance with the literature, as more than 98% of MPA is protein bound, and only a small part is found in PBMCs. It was proved that MPA plasma concentrations reflect better IMPDH activity than MPA concentrations in PBMCs [26]. We found that in our laboratory, chromatographic separation should be performed using the mobile phase buffer at pH 5.20, whereas in Glander et al.’s study [24], the pH of the mobile phase buffer was 5.50 and 5.60 for the method used in centers B and R, respectively. We did not shorten or extend the incubation time of PBMCs. According to the literature, IMPDH activity correlates linearly with the incubation time [24,26]. Due to the lack of symmetry of the XMP peak after 120 min of incubation and no significant difference in XMP production and peak appearance after 180 min of incubation, it was confirmed that the optimal incubation time was 150 min, similar to the Glander et al. study [24]. In Liu et al.’s study [25], we found a similar observation that AMP concentration did not differ between analyzed incubation times, and XMP increased when the samples were incubated up to 180 min. On the contrary, the authors incubated the samples for 120 min with satisfactory experimental operability.

For healthy volunteers, the mean IMPDH activity determined in our study is similar to that described by Liu et al. [25] and almost twofold lower than that presented in Rother et al.’s study [20]. Although there are some other studies concerning healthy volunteers, the difference in units of IMPDH activity causes difficulties in comparing the results.

The range of MPA C_trough_ found in our study was similar to pediatric renal transplant recipients in the early post-transplant period [20]; however, we observed concomitantly twofold lower median A_trough_. In pediatric renal transplant recipients, minimal enzyme activity reflected MPA C_max_ in plasma [15]. In our study, for children with nephrotic syndrome, the lowest IMPDH activity was A_1_, and at this time point, the MPA concentration was the highest. It shows that IMPDH activity reflects MPA concentrations also in children with nephrotic syndrome, as MPA C_max_ generally occurs 1–2 h after MMF administration [1]. On the other hand, if compared to adult renal transplant recipients [27], median A_trough_ and A_2_ were 1.5–2-fold higher in children with nephrotic syndrome, whereas A_1_ was comparable.

Although the method is labor intensive and requires much time for sample preparation, our results suggest that determining IMPDH activity may be applied for assessing the pharmacodynamic effect of MMF therapy in children with nephrotic syndrome. Our results showed that although the lowest IMPDH activity was observed with the highest MPA concentration, target IMPDH activity values remain to be defined. The MPA pharmacokinetics differs between treatment indications, but its influence on IMPDH activity in pediatric patients with nephrotic syndrome is still unclear.

## 4. Materials and Methods

### 4.1. Materials

All solvents and reagents were of HPLC or analytical grade. Adenosine monophosphate (AMP) and IMP were purchased from Santa Cruz Biotechnology (Dallas, TX, USA) whereas XMP was from Jena Bioscience (Jena, Germany). Histopaque^®^-1077, sodium dihydrogen phosphate, NAD^+^, potassium carbonate, and MPA were obtained from Sigma-Aldrich (Saint Louis, MO, USA), whereas potassium chloride was obtained from POCH (Gliwice, Poland) and perchloric acid from Merck (Darmstadt, Germany). Methanol (J. T. Baker, Deventer, Netherlands), tetrabutylammonium bromide (TBAB) (Sigma-Aldrich, Saint Louis, MO, USA), and potassium dihydrophosphate (Merck, Darmstadt, Germany) were used for preparing the mobile phase. Full blood from six healthy volunteers was purchased from the Regional Blood Donation and Blood Treatment Center in Poznań, Poland.

### 4.2. Chromatographic Conditions and Apparatus

The analyses were performed in the HPLC system HP1100 (Hewlett Packard, Palo Alto, CA, USA). Chromatographic separation was done using a Zorbax Eclipse XDB C18 column (150 × 4.6 mm, 5 μm, Agilent Inc., Santa Clara, CA, USA). The mobile phase consisted of methanol and buffer (7 mmoL/L tetrabutylammonium bromide and 50 mmoL/L potassium dihydrophosphate with a pH of 5.2) mixed at the ratio of 90:10 (*v/v*). The flow rate was 1 mL/min. The column was kept at 40 °C, the volume of injection was 25 µL, and the detection was at 254 nm. AMP and XMP peak surface areas were further used for calculating IMPDH activity in PBMCs.

### 4.3. AMP and XMP Concentration

A stock solution of 10 mmoL/L AMP was prepared by dissolving the appropriate amount of the compound in HPLC-grade water. The stock solution of XMP was purchased at a concentration of 10 mmoL/L. The calibration standards were prepared from stock solutions by dilution. For AMP and XMP, the calibration standards were as follows: 1.8, 2.7, 3.6, 9.0, 18.0, 36.0, 90.0, and 180.0 μmoL/L.

For calibration curves, we used 50 μL of calibration standards containing AMP and XMP and added them to 130 µL of calibration curve incubation buffer, which consisted of 40 mmoL/L sodium dihydrogen phosphate and 100 mmoL/L potassium chloride with a pH of 7.4. The resulting concentrations of AMP and XMP were 0.50, 0.75, 1.0, 2.5, 5.0, 10.0, 25.0, and 50.0 μmoL/L. Subsequently, the samples for calibration curves and biological samples were incubated for 120, 150, and 180 min at 37 °C and moderately shaken at 800 rpm. The incubation was ended by adding 20 µL of 4 moL/L perchloric acid, then mixed, placed on ice, and subsequently centrifuged at 14,000× *g* for 5 min at 4 °C. The volume of 170 μL of supernatant was transferred into an Eppendorf Tube^®^, which contained 10 µL of 5 moL/L potassium carbonate for samples neutralization. The content of the tube was mixed and stored at −25 °C for 30 min. After thawing and centrifuging, the supernatant was placed in vials and analyzed using the HPLC method.

### 4.4. Method Validation

The elaborated methods were validated according to the guidelines on the bioanalytical method validation of the European Medicines Agency [28].

The calibration curves for AMP and XMP were built as the relation between peak surface area and theoretical concentration, covering the range of 0.5–50 μmoL/L. Student’s *t* test and Mandel’s fitting test were applied for the evaluation of the linearity. The correlation coefficient *r* was calculated for both curves. Within-run precision and accuracy were assessed based on five samples for four different concentrations: 0.5 µmoL/L (lower limit of quantification—LLOQ), 1.0 µmoL/L (low QC), 25.0 µmoL/L (medium QC), and 50.0 µmoL/L (high QC). Between-run precision and accuracy were assessed based on calibration curves performed within five days. Precision was defined as the CV, and accuracy was expressed as %RE, that is, calculated as the difference (in %) between the mean determined concentration and the nominal concentration.

To check whether MPA was detected under the established HPLC conditions, we analyzed two MPA concentrations (0.5 and 20 μg/mL). These solutions were prepared using the methanol stock solution, which was subsequently diluted with water. The same concentrations of MPA in incubation buffer as a solvent were also analyzed.

### 4.5. In Vivo Application

The modified method was applied to determine IMPDH activity in PBMCs collected from healthy adults and children with nephrotic syndrome treated with MMF.

#### 4.5.1. PBMC Isolation

The method for PBMC isolation was based on the study of Glander et al. [24]. In short, 2.5 mL of blood was diluted with 2.5 mL of phosphate-buffered saline (PBS), then transferred slowly into a falcon tube with 5 mL of Histopaque^®^-1077 and centrifuged at 1200× *g* at room temperature for 20 min. The PBMC layer was collected, washed with 5 mL of PBS, and centrifuged for 5 min at 1200× *g*. The procedure of washing and centrifuging was repeated three times. The cell pellet was resuspended in 250 mL of cold HPLC-grade water (4 °C) and frozen at –80 °C. After thawing, the suspension was centrifuged at 1000× *g* for 2 min, and the supernatant was used for analysis of AMP intracellular concentration and XMP produced in the enzymatic reaction. Protein concentrations were not determined.

#### 4.5.2. Healthy Volunteers

Blood samples were collected from four males and two females aged 38–46 (mean 42 ± 3 years). The volunteers had given their written permission for the use of blood for scientific studies to validate the IMPDH activity assay method. From healthy volunteers, 10 mL of whole blood was collected into EDTA tubes, divided into four tubes, and used for PBMC isolation.

#### 4.5.3. Children with Nephrotic Syndrome Treated with MMF

The analytical method was applied in 12 children (five girls, seven boys) aged 4–16 years (mean age 8 ± 4 years) with nephrotic syndrome who were treated with MMF in the Department of Pediatric Nephrology and Hypertension at Poznan University of Medical Sciences (Poznań, Poland). The children received MMF orally (capsule, tablet, or suspension) in 12 h intervals at the same morning and evening dose (mean dose 473 ± 113 mg BID, range 250–600 mg). The children received MMF from 1 up to 25 months (mean 10 ± 8 months). In three children, glucocorticoids were coadministered during MMF therapy. In no child was proteinuria observed on the day of blood collection.

From children with nephrotic syndrome, four blood samples (3 mL each) were collected into EDTA tubes as follows: before MMF morning dose and 1, 2, and 4 h afterward. For children with nephrotic syndrome, the volume of 0.5 mL of plasma was transferred into another EDTA tube and centrifuged to obtain plasma for MPA determination. Plasma samples were stored at –20 °C until analyzed. MPA plasma concentrations were determined using the HPLC-UV method described elsewhere [12,29] in samples collected before MMF morning dose (C_trough_) and 1 h (C_1_), 2 h (C_2_), and 4 h (C_4_) afterward. The remaining 2.5 mL of blood was used for PBMC isolation for determining IMPDH activity.

All procedures performed in the studies involving human participants were in accordance with the ethical standards of the institutional and national research committee and with the 1964 Helsinki Declaration and its later amendments or comparable ethical standards. The study was approved by the Bioethical Committee at Poznan University of Medical Sciences. Informed consent was obtained from the parents or guardians prior to initiating the study.

#### 4.5.4. AMP and XMP Determination and IMPDH Activity Calculations

For determining AMP and XMP in samples collected from healthy adults and children with nephrotic syndrome, the volumes of 50 μL of PBMC lysates were added to 130 μL of PBMC analysis incubation buffer. This buffer consisted of 1 mmoL/L IMP, 1 mmoL/L NAD^+^, 40 mmoL/L sodium dihydrogen phosphate, and 100 mmoL/L potassium chloride with a pH 7.4.

The IMPDH activity was calculated according to the formula found in Glander et al.’s study [24] using the XMP concentration produced after the enzymatic reaction and incubation with the excess of IMP and NAD^+^. Intracellular AMP concentrations, determined during the same HPLC analysis, were used for normalization of XMP concentrations. The unit of IMPDH activity was µmoL·s^−1^·moL^−1^AMP. For children with nephrotic syndrome, the IMPDH activities were determined before MMF next dose (A_trough_) and 1 h (A_1_), 2 h (A_2_), and 4 h (A_4_) afterward.

#### 4.5.5. Calculations and Statistical Analysis

Based on the linear trapezoidal rule, the area under the effect–time curve from 0 to 4 h (AEC_0–4_) and the area under the concentration–time curve from 0 to 4 h (AUC_0–4_) were calculated for IMPDH activity and MPA concentrations, respectively. IMPDH minimal activity (A_min_), time to reach A_min_ (t_min_), MPA maximal concentration (C_max_), and time to reach C_max_ (t_max_) were extracted from determined IMPDH activities and MPA concentrations. The coefficient of variation (CV) was used to express the variability of pharmacokinetic parameters.

All statistical tests were performed using Statistica software version 13.0 (StatSoft, Inc., Tulsa, OK, USA). Normality was determined by the Shapiro–Wilk test. The differences between variables were estimated using Student’s *t* test and the Mann–Whitney test for normally and non-normally distributed data, respectively. In the case of unequal variations, Cochran’s C test was applied. A *p*-value equal to or lower than 0.05 was considered significant. The results are presented as mean ± standard deviation (SD).

## 5. Conclusions

The modified method is accurate, precise, and linear in the concentration range of AMP and XMP of 0.50–50.0 μmoL/L and may be applied to IMPDH activity determination in children with nephrotic syndrome treated with MMF. IMPDH activity should be determined after one thawing of PBMCs due to the change in AMP and XMP concentrations after subsequent thawing. Under the conditions of analysis, the MPA peak did not occur during the determination of AMP and XMP. For children with nephrotic syndrome, IMPDH activity was variable within 4 h after MMF administration, and the lowest IMPDH activity was observed concomitantly with the highest MPA concentration. Determination of IMPDH activity might be considered as a biomarker of MMF treatment in children with nephrotic syndrome; however, the analytical method is labor intensive and requires much time, and the target values are still to be defined.

## Figures and Tables

**Table 1:** The freezing–thawing stability of AMP, XMP, and inosine 5′-monophosphate dehydrogenase (IMPDH) activity determined in healthy volunteers.

	AMP (μmoL/L)	XMP (μmoL/L)	IMPDH Activity (µmoL∙s^−1^∙moL^−1^AMP)
**Volunteer No. 3**			
I cycle	16.24	5.49	37.54 (100%)
II cycle	16.23	4.20	28.78 (77%)
III cycle	17.94	6.29	38.98 (104%)
**Volunteer No. 4**			
I cycle	16.73	7.74	51.41 (100%)
II cycle	14.42	6.21	47.84 (93%)
III cycle	13.09	8.48	72.02 (140%)
**Volunteer No. 5**			
I cycle	9.21	3.58	43.12 (100%)
II cycle	8.27	2.89	38.82 (90%)
III cycle	7.11	2.52	39.32 (91%)

AMP, adenosine monophosphate; IMPDH, inosine 5′-monophosphate dehydrogenase; XMP, xanthosine monophosphate.

**Table 2:** AMP and XMP concentrations and calculated IMPDH activity in children (*n* = 12) with nephrotic syndrome treated with MMF.

	**AMP (μmoL/L)**			
Time (h)	0	1	2	4
mean	24.61	21.27	18.51	22.18
SD	17.50	11.78	13.49	16.04
min	3.46	3.99	2.98	2.87
max	60.03	41.60	42.14	54.59
	**XMP (μmoL/L)**			
Time (h)	0	1	2	4
mean	5.98	2.74	2.79	5.04
SD	3.57	2.07	2.37	3.42
min	0.80	0.54	0.39	2.21
max	13.07	5.80	7.59	13.22
	**IMPDH Activity (µmoL∙s^−1^∙moL^−1^AMP)**			
Time (h)	0	1	2	4
mean	39.2	25.6	25.4	38.5
SD	27.4	29.4	17.6	26.1
min	9.5	2.8	1.7	5.8
max	98.6	96.2	49.6	86.5

AMP, adenosine monophosphate; IMPDH, inosine 5′-monophosphate dehydrogenase; MMF, mycophenolate mofetil; XMP, xanthosine monophosphate.

**Table 3:** Inosine 5′-monophosphate dehydrogenase (IMPDH) pharmacodynamic and mycophenolic acid (MPA) pharmacokinetic parameters in children with nephrotic syndrome treated with mycophenolate mofetil (MMF).

	**IMPDH A_trough_** **(µmoL∙s^−1^∙moL^−1^ AMP)**	**IMPDH A_min_** **(µmoL·s^−1^·moL^−1^ AMP)**	***t*_min_** **(h)**	**IMPDH AEC_0–4_** **(h∙µmoL·s^−1^·moL^−1^ AMP)**
mean	39.2	18.0	1	121.8
SD	27.4	15.2	1	88.4
min	9.5	1.7	0	23.6
max	98.6	49.6	2	293.9
	**MPA C_trough_** **(μg/mL)**	**MPA C_max_** **(μg/mL)**	**t_max_** **(h)**	**MPA AUC_0–4_** **(h μg/mL)**
mean	1.84	10.59	1	22.59
SD	1.17	2.97	0	7.34
min	0.39	5.28	1	12.21
max	4.34	13.98	2	35.31

A_trough_, activity in the sample collected before the next drug dose administration; A_min_, minimal enzyme activity; AMP, adenosine monophosphate; AEC_0–4_, area under the effect–time curve from 0 to 4 h; AUC_0–4_, area under the concentration–time curve; C_trough_, concentration before the next drug dose administration; C_max_, maximal concentration, IMPDH, inosine 5′-monophosphate dehydrogenase; MPA, mycophenolic acid; MMF, mycophenolate mofetil; t_max_, time to reach C_max_; t_min_, time to reach A_min_.
